# In silico drug repositioning against human NRP1 to block SARS-CoV-2 host entry

**DOI:** 10.3906/biy-2012-52

**Published:** 2021-08-30

**Authors:** Şeref GÜL

**Affiliations:** 1 Department of Chemical and Biological Engineering, Koç University, İstanbul Turkey; 2 Biotechnology Division, Department of Biology, Faculty of Science, İstanbul University, İstanbul Turkey

**Keywords:** SARS-CoV-2, COVID-19, NRP1, drug repositioning, eltrombopag, sitagliptin

## Abstract

Despite COVID-19 turned into a pandemic, no approved drug for the treatment or globally available vaccine is out yet. In such a global emergency, drug repurposing approach that bypasses a costly and long-time demanding drug discovery process is an effective way in search of finding drugs for the COVID-19 treatment. Recent studies showed that SARS-CoV-2 uses neuropilin-1 (NRP1) for host entry. Here we took advantage of structural information of the NRP1 in complex with C-terminal of spike (S) protein of SARS-CoV-2 to identify drugs that may inhibit NRP1 and S protein interaction. U.S. Food and Drug Administration (FDA) approved drugs were screened using docking simulations. Among top drugs, well-tolerated drugs were selected for further analysis. Molecular dynamics (MD) simulations of drugs-NRP1 complexes were run for 100 ns to assess the persistency of binding. MM/GBSA calculations from MD simulations showed that eltrombopag, glimepiride, sitagliptin, dutasteride, and ergotamine stably and strongly bind to NRP1. In silico Alanine scanning analysis revealed that Tyr^297^, Trp^301^, and Tyr^353^ amino acids of NRP1 are critical for drug binding. Validating the effect of drugs analyzed in this paper by experimental studies and clinical trials will expedite the drug discovery process for COVID-19.

## 1. Introduction

Severe acute respiratory syndrome coronavirus 2 (SARS-CoV-2) infected over 40 million people and resulted in more than 1.5 million deaths around the world since December 2019.^1^Worldometers (2020). COVID-19 Coronavirus Pandemic [online]. Website: https://www.worldometers.info/coronavirus/ [accessed 05 December 2020]. The coronavirus disease-2019 (COVID-19) has symptoms of pneumonia manifesting fever, fatigue, dyspnea, chest pain, and cough which are also observed in other viral respiratory diseases (Yi et al., 2020). The whole-genome analysis showed that SARS-CoV-2 belongs to the *Betacoronovirus* genera and has a 96% genomic sequence identity to that of bat coronavirus (Wu et al., 2020b; Zhou et al., 2020). Despite the high genomic similarities, SARS-CoV-2 behaves more aggressively than SARS-CoV in terms of viral load, reaching the peak RNA concentration, tissue tropism, and transmission efficiency (Wolfel et al., 2020). 

SARS-CoV-2 genome is composed of a (+) sense single-strand RNA (ssRNA) with 14 open reading frames (ORFs). SARS-CoV-2 synthesizes two polyproteins, ORF1a and ORF1ab, and four structural proteins e.g., spike (S), envelop (E), matrix (M), and nucleocapsid (N) proteins and accessory proteins (3a, 3b, p6, 7a, 7b, 8b, 9b, and orf14) (Wu et al., 2020a; Yoshimoto, 2020). Proteolytic cleavage of ORF1a and ORF1ab by proteases leads to the production of 16 nonstructural proteins (nsps). Among nsps, nsp5 (3C-like protease, 3CL^pro^) first cleaves its bounds with nsp4 and nsp6, then cleaves the ORF1a and ORF1ab at 11 sites (Du et al., 2004; Muramatsu et al., 2013). Besides 3CL^pro^, nsp3 (papain-like protease, PL^pro^) cleaves these polyproteins at three sites and completes the production of 16 nsps (Baez-Santos et al., 2015; Thiel et al., 2003). 

S protein of SARS-CoV-2 interacts with angiotensin-converting enzyme 2 (ACE2) that has been accepted as the main receptor for the entry of the virus (Wang et al., 2020; Yan et al., 2020). ACE2, a membrane protein, is expressed in lung, upper and stratified epithelial cells of the esophagus, heart, kidney, testis, colon, ileum, and intestine (Donoghue et al., 2000; Zhang et al., 2020). Despite the attenuated expression of ACE2 in elderly people, increased or unaffected severity of COVID-19 symptoms (Walls et al., 2020) in these people (Liu et al., 2020; Omori et al., 2020; Singh et al., 2020a), and high viral loads in the throat (Wolfel et al., 2020) suggest the existence of an alternative receptor for the virus entry. 

A polybasic sequence (Arg^682^Arg-Ala-Arg^685^) at the S1/S2 region of S protein of the SARS-CoV-2 may explain its higher transmission rate and tissue tropism. This sequence is also conserved in the S protein of various pathogenic human viruses such as Ebola, influenza, and HIV-1 (Tse et al., 2014). Cleavage of the S protein within the polybasic sequence, which is primed by transmembrane serine protease 2 (TMPRSS2) via furin produces S1 and S2 (Hoffmann et al., 2020b). *FURIN* mediated cleavage of S protein results in increased tropism and the infection rate of SARS-CoV-2, most probably because of the formation of new cell surface binding regions (Hoffmann et al., 2020a; Wrapp et al., 2020). Knocking down *FURIN* gene or using its inhibitor decreases the virus entry to cells and syncytia formation in the infected cells, respectively (Shang et al., 2020; Walls et al., 2020). 

(Arg^682^Arg-Ala-Arg^685^) sequence is aligned with [R/K]XX[R/K] motif (R: arginine, K: lysine, and X is any amino acid), named as C-end rule (CendR). CendR binds and activates cell surface receptors neuropilin (NRP1,2) (Teesalu et al., 2009). NRP1 binds to several growth factors such as vascular endothelial growth factors (VEGFs) (Soker et al., 1998), hepatocyte growth factor (Sulpice et al., 2008), and transforming growth factor (Cao et al., 2010). Among those, the interaction between NRP1 and VEGF-A_165_ has been extensively studied ( Teesalu et al., 2009; Jarvis et al., 2010; Parker et al., 2012; Fantin et al., 2014; Jia et al., 2014; Mota et al., 2018; Powell et al., 2018). C-terminal arginine residue and CendR motif of VEGF-A_165_ critically interact with b1 domain of NRP1 (NRP1-b1) in which natural and artificial NRP1 inhibitors were explored (Vander Kooi et al., 2007). Having a cavity on CendR binding pocket of NRP1 made it an attractive target for designing small-molecules. Several molecules blocking NRP1-VEGF interaction were developed (Jarvis et al., 2010; Powell et al., 2018). First designed NRP1 inhibitor, EG00229, occupied the VEGF-A binding region by directly interacting with Tyr^297^, Glu^348^, Thr^349^, and Tyr^353^ (Jarvis et al., 2010). Based on the discovered scaffold of EG00229, a more potent molecule, EG01377 was developed (Powell et al., 2018). Both these molecules bind to NRP1 with a very similar mode. 

Recent studies showed that interaction between NRP1 and protein S of SARS-CoV-2 is required for the SARS-CoV-2 cell entry (Cantuti-Castelvetri et al., 2020; Daly et al., 2020). While coexpression of NRP1 with ACE2 and TMRPSS2 increased the infection, monoclonal antibody targeting the b1b2 extracellular domain of NRP1 significantly inhibited the infection. The stimulating effect of NRP1 on the virus infection diminished when the furin cleavage site of SARS-CoV-2 is mutated (Cantuti-Castelvetri et al., 2020). Analysis of direct interaction between CendR peptide of protein S and NRP1 using isothermal titration calorimetry method showed that binding affinity of the complex is between 20 and 13µM depending on the pH. Mutating the critical arginine residue (Arg^685^) on the CendR of protein S prevented the NRP1 binding (Daly et al., 2020). The crystallographic analysis confirmed that the binding mode of protein S’ CendR to the NRP1 is similar to the previously resolved NRP1-VEGF-A structure (Daly et al., 2020; Parker et al., 2012). 

To date, there is no consensus on the treatment of COVID-19 yet. Since developing a new drug is costly and requiring a long period, repurposing of U.S. Food and Drug Administration (FDA) approved drugs may provide an alternative approach to find a therapeutic for the COVID-19 treatment within a reasonable time and cost. Multiple targets are available to neutralize COVID-19. For example, virus entry proteins on the host (ACE2, TMPRSS2, NRP1), replication machinery of the virus (3CL^pro^ RNA-dependent RNA polymerase), virus proteins taking roles on the assembly mechanism (protein E) and the release of the virus (protein M and N) are candidates for drug development and drug repurposing ( Venkatagopalan et al., 2015; Schoeman and Fielding, 2019; Li et al., 2020a; Sarma et al., 2020). Given the crucial role of NRP1 for the SARS-CoV-2 entry into the host specifically in elderly people, this in silico study aimed to repurpose FDA-approved drugs against b1 domain of NRP1 to find a promising drug to block SARS-CoV-2 infection. For this purpose, initially docking simulations were run to find drugs with high binding affinity to b1 domain of NRP1. As a control, previously identified NRP1 inhibitor, EG01377, was docked to the same pocket. While docking binding energy of the inhibitor is around –6.0 kcal/mol, over 250 drugs have –7.4kcal/mol or lower AutoDock Vina binding energy. Drugs were sorted based on docking binding energies, and an extensive literature search was done to select well-tolerated drugs for further analysis. One hundred ns molecular dynamics simulations (MD) of drug-NRP1 complexes showed that thrombopoietin receptor agonist eltrombopag, migraine drug ergotamine, drugs for type 2 diabetes sitagliptin and glimepiride, and antiandrogen dutasteride can stably interact with NRP1. Crystal structure of NRP1 in complex with EG01377 (PDB ID: 6FMF) was simulated to compare the affinity of selected drugs with its inhibitor. Binding free energy calculation using MM/GBSA method showed that selected drugs have a comparable or better affinity to NRP1 than EG01377. Alanine scanning calculations revealed Tyr^297^, Trp^301^, and Tyr^353^ as the critical amino acid residues for drug-NRP1 interaction. These findings may be used in experimental studies and clinical trials to test the effect of promising drugs alone or in combination with current COVID-19 treatment protocols.

## 2. Materials and methods

High-resolution human NRP1 structure (PDB ID: 6FMC) was retrieved from The Protein Data Bank^2^RCSB PDB (Research Collaboratory for Structural Bioinformatics PDB) (2021). The Protein Data Bank [online]. Website www.rcsb.org [accessed 18 September 2020]. (Powell et al., 2018). Protein was prepared for docking simulations via Dock Prep module of UCSF Chimera as described previously ( Tardu et al., 2016; Doruk et al., 2020; Gul et al., 2020). Crystal water molecules were removed and if alternate locations are available for residues, the ones with the higher occupancies were selected. Rotamer library developed by Shapovalov and Dunbrack (2011) was used to complete missing side chains of amino acids, polar hydrogens were added, and nonpolar hydrogens were merged with bound atoms. Atom charges should be defined to run docking simulations. Thus, Gasteiger charges to each atom in the protein were assigned using Auto Dock Tools (ADT) (v. 1.5.6). Structures of drugs were downloaded from Zinc15 database catalogue of FDA-approved drugs. Set of drugs used in this study were imported from the U.S. Environmental Protection Agency’s (EPA) distributed structure-searchable toxicity (DSSTox) database. The 3948 drugs and NRP1 inhibitor (EG01377) were prepared for docking by using ADT suite. Inhibitor binding pocket of NRP1 was targeted for docking simulations in which grid center was placed on the center of Tyr ^297^, Glu^348^, and Tyr^353^ side chains with 6400 Å^3^of the grid box. AutoDock Vina (version 1.1.2) was used for the docking (Trott and Olson, 2010).

With the aid of VMD, NRP1 structure (PDB ID: 6FMF) was solvated using TIP3P water molecules in a rectangular box with edge lengths of x = 75 Å, y = 80 Å, z = 75 Å and a size of 4.50 × 10>^5^Å^3^. Solvated protein was neutralized and then ionized with sodium-chloride salt (Na^+^ and Cl^-^) to 150 mM final concentration to mimic the physiological conditions (Humphrey et al., 1996). Twenty thousand steps of energy minimization (via conjugate gradient) was performed. The minimized system was gradually heated and then equilibrated for 1.4 ns (NPT ensemble) with constraints on the protein. Constrains starting from 2 kcal/mol/Å^2^ were reduced by 0.5 kcal/mol/Å^2^ for each 0.4 ns equilibration run. Production simulation of 100 ns for each equilibrated drug-NRP1 complex was run using 2 fs time step at 310K and 1atm pressure. Langevin thermostat and Langevin barostat maintained the temperature and pressure, respectively. To calculate the force acting on the system van der Waals (12Å cut-off) and long-range electrostatic interactions (via particle-mesh Ewald) were calculated. NAMD (Phillips et al., 2005) software and CHARMM36m force field (Huang et al., 2017) were used for all MD simulations. CHARMM-GUI server was used to generate parameters of drugs (Jo et al., 2008; Kim et al., 2017). 

Analyses of protein-drug interactions were done by following these steps: 1) MD trajectory of each drug-protein simulation was visualized to inspect the position of the drug. If the drug leaves the binding pocket, that one is eliminated. 2) For simulations that drug-protein interaction was maintained for 100 ns, root mean square deviation (RMSD) of C_α_ atoms was calculated to verify the successful equilibration of the system. 3) Contact frequency between drug and nearby amino acid residues were carried out for determining the binding residues. 4) Binding free energy (BFE) of drugs was calculated using molecular mechanics generalized born surface area (MM/GBSA) method. 5) Amino acid residue with a high contact frequency was mutated to Alanine to calculate their contribution to BFE of drugs. All MD analyses were performed and protein figures were prepared by using VMD and Pymol, respectively (Humphrey et al., 1996; DeLano, 2009). RMSD trajectory tool and timeline function in VMD were used to calculate RMSD of protein and contact frequencies between drug and protein, respectively. Unstructured and highly dynamic first 9 N-terminal amino acid residues were excluded from the RMSD calculation. 

For MM/GBSA and Alanine scanning calculations, MMPBSA.py script of AmberTools20 was used (Case et al., 2020). For each calculation, 25,000 frames from 100 ns simulations were used. BFE between drug and protein is calculated based on Equation 1:

ΔG_binding_ = G_complex_ – G_receptor_ – G_ligand_ (1)

G_complex_ : Energy of protein-drug complex, 

G_receptor_ : Energy of protein only, 

G_ligand_ : Energy of unbound drug.

## 3. Results

To discover an antiviral drug diverse targets are available from viral replication enzymes to host proteins facilitating the virus entry and to proteins responsible for the virus release (Boopathi et al., 2020; Lo et al., 2020; Wu et al.,2020b). Several in silico and in vivo drug repurposing studies are carried out using proteases (3CL^pro^ and PL^pro^) and RNA-dependent RNA polymerase (RdRp) of SARS-CoV-2 as targets (Bharadwaj et al., 2020; Ghosh et al., 2020; Gul et al., 2020; Li et al., 2020c; Wang, 2020). In addition, host proteins TMPRSS2 and ACE2 which primes protein S and mediates SARS-CoV-2 entry to host, respectively, are targeted for drug repositioning as well (Bagheri and Niavarani, 2020; Busnadiego et al., 2020; Carino et al., 2020; Choudhary et al., 2020; DurdaGi, 2020; Kumar et al., 2020; Singh et al., 2020b). 

**Figure 1:**
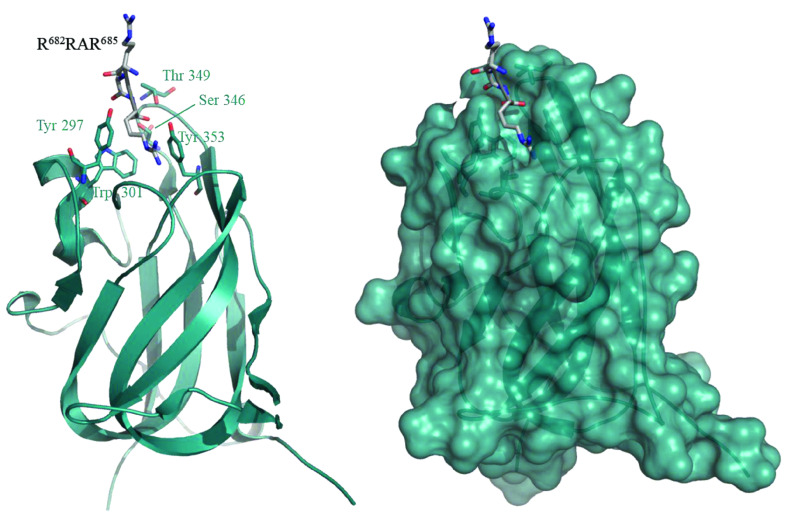
Structure of NRP1 in complex with polybasic peptide at the C-terminal of protein S (PDB ID: 7JJC). NRP1 is shown in cartoon at left critical residues are shown in ball and sticks (cyan color), polybasic peptide is shown in ball and sticks (gray color) representation. NRP1 is shown in surface representation at right.

NRP1 is a recently identified receptor that facilitates the SARS-CoV-2 cell entry in coordination with ACE2 and TMPRSS2 (Cantuti-Castelvetri et al., 2020; Daly et al., 2020). S protein of SARS-CoV-2 attaches to the host cells for the virus entry (Wang et al., 2020; Yan et al., 2020). When the S protein is cleaved by the host protease, S1 and S2 proteins are generated. S1 has a polybasic sequence (Arg^682^-Arg-Ala-Arg^685^) at the C-terminal which interacts with b1 domain of NRP1 (Cantuti-Castelvetri et al., 2020; Daly et al., 2020) (Figure 1). Mutating the critical Arg^685^ diminishes protein S and NRP1 binding (Daly et al., 2020) and antibody against b1b2 domain of NRP1 relieve the SARS-CoV-2 infection (Cantuti-Castelvetri et al., 2020). Drugs having a high affinity to CendR binding pocket of NRP1 can be used in the COVID-19 treatment by blocking SARS-CoV-2 entry into cells. 

### 3.1. Docking simulations

**Figure 2:**
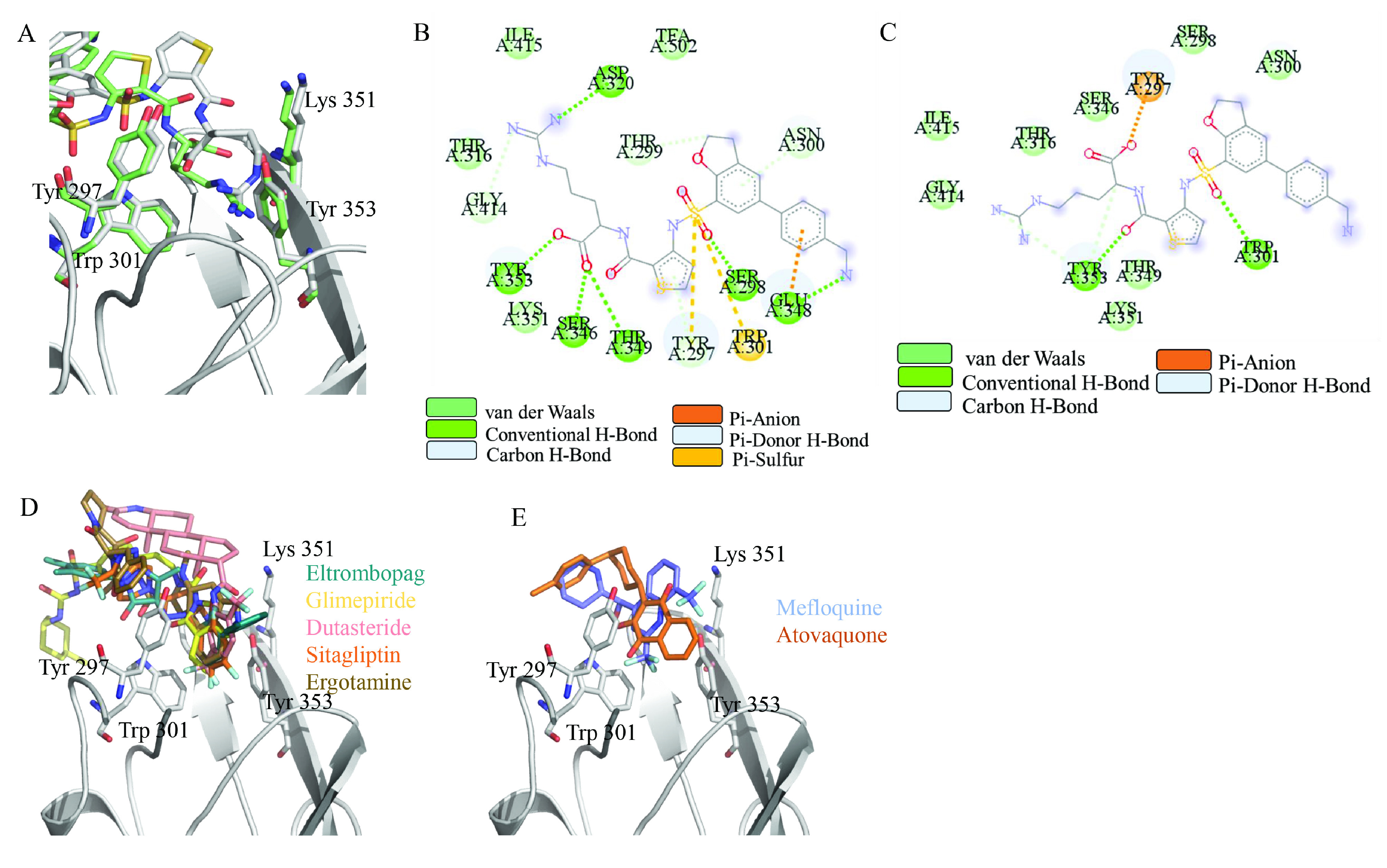
(A) Binding of EG01377 (shown in sticks representation) in the crystal (PDB ID: 6FMF) (carbon atoms are shown in green) in the docking simulation (carbon atoms are shown in white) to b1 domain of NRP1 (shown in white cartoon representation). (B) 2D interaction diagram of EG01377 binding to NRP1 in crystal structure (6FMF). (C) 2D interaction diagram of EG01377 in docking simulation. Diagrams were generated by using Discovery Studio Visualizer. (D) Binding mode of eltrombopag, glimepiride, dutasteride, sitagliptin, and ergotamine to NRP1 obtained from the docking. (E) Binding of two antimalarial drugs mefloquine and atovaquone to NRP1 obtained from the docking.

AutoDock Vina program was used to calculate binding energy and predict the binding mode of drugs to the target pocket as described before (Tardu et al., 2016; Gul et al., 2020). During the docking simulations, receptor (NRP1) was treated as a rigid body and drugs were allowed to sample different conformations in the CendR binding pocket. NRP1 inhibitor, EG01377 (Powell et al., 2018), was docked to NRP1 as a control to evaluate the binding affinities of drugs against NRP1. Vina binding energy of EG01377 was calculated as –6.0 kcal/mol (Table 1). The docked conformation of EG01377 is in contact with Tyr^297^, Trp^301^, Lys^351^, and Tyr^353^ similar to observed in the crystal structure (6FMF) (Figure 2A). Docking simulations revealed that over 100 molecules have Vina binding energies –7.6 kcal/mol or lower (Table S1). Histogram representation of Vina binding energies of all drugs are provided in (Figure S1). Top 15 of those drugs having the Vina binding energies between –8.5 and –8.0 kcal/mol were given in (Table 1) with their ZINC IDs, structures, and types. 

Docking simulations showed that, according to the descriptions in DrugBank (Wishart et al., 2006), various types of drugs such as anticancer, antipsychotics, antiinflammatory, antibiotics, antidiabetics, and estrogen hormone may bind to NRP1 (Table S1). For further analysis, widely used drugs eltrombopag (–8.5 kcal/mol), glimepiride (–8.2 kcal/mol), dutasteride (–8.2 kcal/mol), sitagliptin (–8.2 kcal/mol), ergotamine (-8.1 kcal/mol) at the top of the docking result list, and two antimalarial drugs, mefloquine (–8.0 kcal/mol) and atovaquone (–7.9 kcal/mol), having similar binding energies to top drugs were selected. Two-dimensional diagram of interaction pattern of EG01377 in the crystal showed that it can generate hydrogen bond, or interact via van der Waals or pi interactions with Asp^320^, Ile^415^, Thr^316^, Gly^414^, Tyr^353^, Lys^351^, Ser^346^, Thr^349^, Tyr^297^, Ser^298^, Trp^301^, Glu^348^, and Asn^300 ^(Figure 2B). Among those, hydrogen bond and pi interactions between the inhibitor and Tyr^297^, Tyr^353^, and Trp^301^; and van der Waals interactions generated through Ser^298^, Ser^346,^ Thr^316^, Ile^415^, Gly^414^, Thr^349^, Lys^351^, and Asn^300^ were reproduced in docking pose as well. However, some hydrogen bond and pi interactions between the inhibitor and amino acids such as inhibitor-Asp^320^ and Glu^348^ in the crystal could not be observed in the docking pose. Hydrogen bond interactions between the inhibitor and Ser^298^, Thr^349^, Ser^346^ in the crystal were generated as van der Waals type interaction in docking conformation (Figure 2C). Note that to compare docking position of EG01377 with that of in crystal, structure with 6FMF PDB ID was used since the binding mode of inhibitor in 6FMF was stated as free of artifact in the original study (Powell et al., 2018). To solidify the idea of using NRP1 inhibitor as an antiviral drug, the binding mode of S protein to NRP1 was analyzed (Daly et al., 2020) and compared with that of EG01377. The peptide of S protein interacts with Asp^320^, Tyr^297^, Trp^301^, Thr^349^, Lys^351^, Tyr^353^, and Ser^346^ of NRP1 via either of hydrogen bond, pi, and van der Waals interactions. Interaction between EG01377 and all of these amino acids shows that NRP1 inhibitor has the potential to be an antiviral drug. Furthermore, the binding poses of selected drugs occupy the pocket surrounded by Tyr^297^, Trp^301^, Lys^351^, and Tyr^353^ in docking simulations which supports the idea of these drugs may behave as NRP1 inhibitors (Figures 2D and 2E). To evaluate the persistency and strength of drug-NRP1 complexes near physiological conditions, MD simulations were run.

**Table 1:** List of top 15 drugs having the best binding affinity to NRP1 inhibitors.

ZINC ID and/ordrug name	2D Structure	Vina binding affinity (kcal/mol)	Type
ZINC000011679756/Eltrombopag	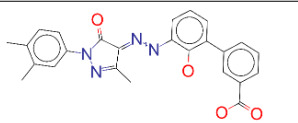	–8.5	Thrombopoietin receptor agonist
ZINC000003830767/Estradiol benzoate	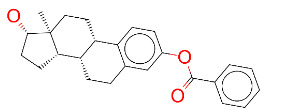	–8.5	Estrogenic steroids
ZINC000064033452/Lumacaftor	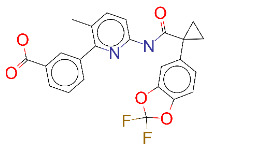	–8.4	Used to treat fibrosis (CF) in patients having homozygous F508del mutation in their CFTR gene.
ZINC000011681563/Netupitant	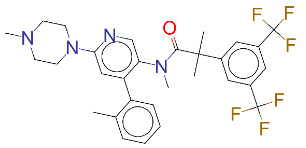	–8.3	Antiemetic
ZINC000000006157/Epinastine	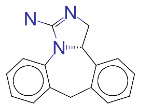	–8.3	Antiallergic
ZINC000000002070/Sorbinil	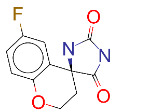	–8.3	Inhibitor of aldose reductase
ZINC000000537791/Glimepiride	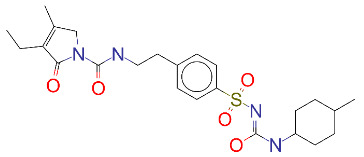	–8.2	Antidiabetics
ZINC000003932831/Dutasteride	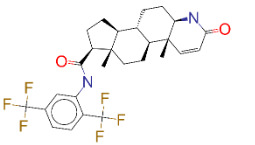	–8.2	Antiandrogen
ZINC000001489478/Sitagliptin	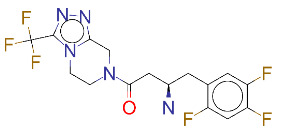	–8.2	Antidiabetics
ZINC000000004724/Trileptal	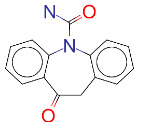	–8.2	Antiepileptic
ZINC000001996117/ Darifenacin	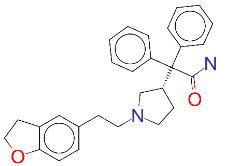	–8.1	Treats urinary incontinence
ZINC000052955754/Ergotamine	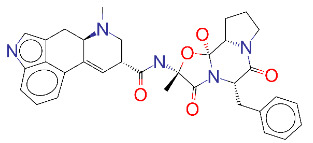	–8.1	Antimigraine
ZINC000003831448/Silibinin	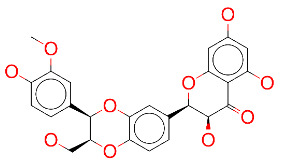	–8.1	Anticancer and various pharmacological effect
ZINC000003978005/Dihydroergotamine	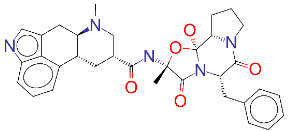	–8.0	Antimigraine
ZINC000003869855/Dicumarol	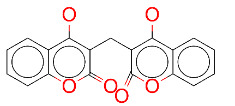	–8.0	Anticoagulant
EG01377	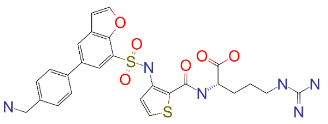	–6.0	Inhibitor of NRP1

### 3.2. MD simulations and binding free energy calculation

**Figure 3:**
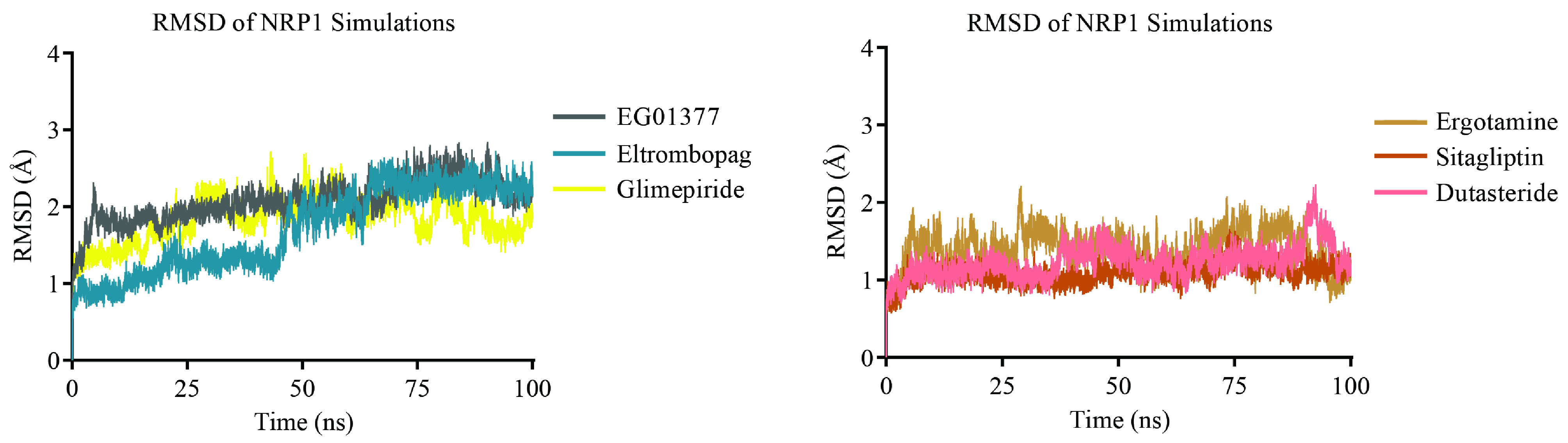
Root mean square deviation (RMSD) of C_α_ atoms in drug-NRP1 simulations.

In short MD simulations (20 ns) eltrombopag, glimepiride, dutasteride, sitagliptin, and ergotamine stably interacted with NRP1, however, atovaquone and mefloquine did not. These two antimalarial drugs left their initial docking positions and moved to the solution even in two independent trials. Then we extended 20 ns simulations for those who could interact with the protein in these short simulations to 100 ns to assess the stability of their interaction with NRP1. In a control simulation, the EG01377-NRP1 complex obtained from crystal (6FMF) was simulated for 100 ns. Visual inspection of long simulations showed that all selected drugs and the inhibitor retained their docking positions on the NRP1 through the entire simulation. Root mean square deviation analysis (RMSD) of C_α_ atoms showed that simulations reached equilibrium around 2Å after an initial jump within 1–2 ns (Figure 3). 

**Figure 4:**
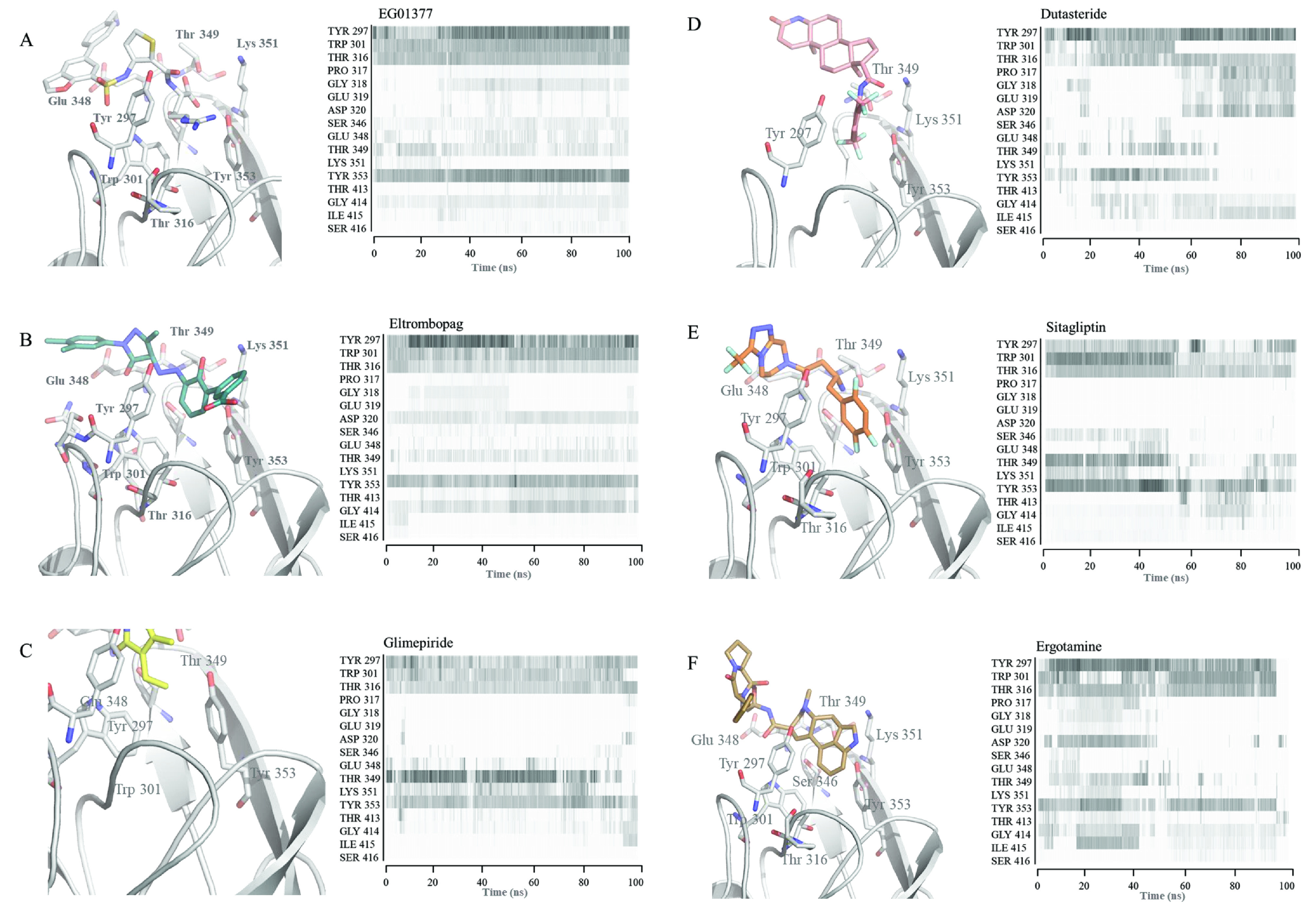
The binding pose in docking simulations and interaction frequency of amino acids from 100 ns MD simulations for NRP1 in complex with (A) EG01377, (B) eltrombopag, (C) glimepiride, (D) dutasteride, (E) sitagliptin, (F) ergotamine.

To examine the interaction between drugs and critical residues, the contact frequency between drugs and selected amino acids around the binding pocket were visualized. Thus, the persistency of drug-amino acid interactions observed in the initial docking position could be assessed (Figures 4A–4F). Docking of EG01377 showed that the inhibitor interacts with Tyr^297^, Trp^301^, Thr^316^, Glu^348^, Thr^349^, Lys^351^, Tyr^353^. In MD simulations, EG01377 interacted frequently with Tyr^297^, Trp^301^, Thr^316^, and Tyr^353^ which have aromatic (Tyr and Trp) and polar (Thr) side chains. In addition to these highly interacting amino acids, the inhibitor interacted with Glu^348^ and Thr^349^ through 100 ns simulation (Figure 4A). As a result, MD simulation of EG01377-NRP1 analysis verified interacting residues in the docking. Contact frequency analysis for eltrombopag-NRP1 simulation indicated that similar to EG01377, Tyr^297^, Trp^301^, Thr^316^, and Tyr^353^ are the frequently interacting residues. The same residues were also identified in docking simulation (Figure 4B). In addition to these highly interacting amino acids, interaction between Asp^320^ and eltrombopag that is identified in the EG01377-NRP1 crystal structure (6FMF) makes eltrombopag drug a prime candidate. One minor difference between EG01377 and eltrombopag simulations is that while EG01377 interacted with Gly^318^, eltrombopag interacted more with Gly^414^. 

Glimepiride and sitagliptin frequently interacted with the same amino acids that are Tyr^297^, Trp^301^, Thr^316^, Thr^349^, and Tyr^353 ^(Figures 4C and 4E) in MD simulations. To note some interactions altered throughout the simulations. For example, interaction with highly interacting amino acids Thr^349^ and Lys^351^ fluctuated as simulations progressed. Despite slight differences in their docking positions, interacting residues in docking simulations are very similar. While glimepiride interacted with Tyr^297^, Trp^301^, Thr^316^, Glu^348^, Thr^349^, and Tyr^353^, sitagliptin fitted a deeper part of the pocket and, in addition to glimepiride’s interacting amino acids, interacted with Thr^316^ (Figures 4C and 4E). Overall, nearby residues observed in docking simulations of glimepiride and sitagliptin were verified by MD simulations.

The docking position of dutasteride showed that the drug interacts with Tyr^297^, Thr^349^, Lys^351^, and Tyr^353^. MD simulation of dutasteride-NRP1 showed that dutasteride behaved less similarly with the inhibitor and did not interact with Trp^301^ and Tyr^353^ after a certain time (Figure 4D). Possibly dutasteride translated in the binding pocket and started to interact with new residues such as Gly^318^, Glu^319^, Asp^320^, and Ile^415^.

Ergotamine showed similar interaction frequency patterns with the inhibitor and constantly interacted with Tyr^297^, Trp^301^, Thr^316^, and Tyr^353^ (Figure 4F). In addition to these, ergotamine interacted with Asp^320^, Thr^349^, and Ile^415^ in certain periods of the MD simulation. In docking simulation ergotamine interacted with Tyr^297^, Trp^301^, Thr^316^, Ser^346^, Glu^348^, Thr^349^, Lys^351^, and Tyr^353^. While some of the amino acids recognized in the docking lost contact with ergotamine such as Ser^346^ and Glu^348^, most of them interacted with ergotamine in the whole MD simulation.

To determine the binding free energy (BFE) between drugs and NRP1 MM/GBSA method was used. The BFE of eltrombopag, glimepiride, dutasteride, sitagliptin, and ergotamine was calculated as –17.11, –12.55, –9.47, –13.34, and –14.95 kcal/mol, respectively (Table 2). BFE of EG01377 was calculated as –16.19 kcal/mol. 

**Table 2:** MM/GBSA BFE of selected drugs and NRP1 inhibitor.

Drug name	MM/ GBSA BFE (kcal/mol)
Eltrombopag	–17.11 ± 2.99
Ergotamine	–14.95 ± 7.40
Sitagliptin	–13.34 ± 3.90
Glimepiride	–12.55 ± 3.48
Dutasteride	–9.47 ± 4.41
EG01377	–16.19 ± 4.20

A well-tolerated drug eltrombopag stimulates platelet synthesis in patients having low blood platelet (Erickson-Miller et al., 2009). In comparison to EG01377, eltrombopag has comparable Vina binding energy (–8.5 kcal/mol) and BFE (–17.11 kcal/mol) calculated from docking and MD simulations, respectively. During the MD simulations interacting residues of EG01377 and eltrombopag with NRP1 are very similar to each other (Figure 4B). A previous drug-repurposing study against 3CL^pro^ and RdRp of SARS-CoV-2 from our group showed that eltrombopag may bind to the active site of 3CL^pro^ and nsp8 binding site of RdRp which may attenuate the virus activity (Gul et al., 2020). Eltrombopag exhibits in vitro antiviral properties with IC_50_ lower than 10µM (Jeon et al., 2020). This report shows that eltrombopag can strongly interact with NRP1, as well. If these in silico findings can be verified by in vitro and cell-culture experiments, eltrombopag can be a very strong antiviral drug by blocking SARS-CoV-2-NRP1 binding to host and alleviating the virus replication.

Two antidiabetics, glimepiride and sitagliptin are well-tolerated drugs used to treat type2 diabetes (Bautista et al., 2003; Raz et al., 2008). Both drugs have the same Vina binding energies (–8.2 kcal) in docking simulations and very similar BFEs calculated from MM/GBSA analysis that are –13.34 and –12.55 kcal/mol for sitagliptin and glimepiride, respectively. Interacting amino acid residues and BFEs of these antidiabetics are quite alike to EG01377 (Figures 4C and 4E). Sitagliptin is an inhibitor of dipeptidyl-peptidase IV (DPP-4) that is the host receptor of the Middle East respiratory syndrome (MERS)-CoV (Karasik et al., 2008; Raj et al., 2013) and in silico studies suggested that protein S of SARS-CoV-2 can bind to DPP4 as well (Li et al., 2020b; Vankadari and Wilce, 2020). Administration of sitagliptin to patients suffering from diabetes and COVID-19 does not only help to lower the blood glucose level but may also reduce the SARS-CoV-2 entry to the cells.

Dutasteride, another well-tolerated drug, inhibits type I and II 5α-reductase and shows antiandrogenic activity (Roehrborn et al., 2002; Andriole and Kirby, 2003). 5α-reductase converts testosterone to dihydrotestosterone and enlarges the prostate glands. Thus, dutasteride is used to treat prostatic conditions by blocking the 5α-reductase activity (Makridakis et al., 2000). Besides the effect on the prostate gland, dutasteride suppresses the transmembrane serine protease 2 (TMPRSS2) expression (Mostaghel et al., 2014) which primes protein S of the SARS-CoV-2 (Hoffmann et al., 2020b). Docking binding energy and MM/GBSA calculations show that dutasteride has a high affinity to NRP1 (Tables 1 and 2). Relatively higher BFE with a high standard deviation of dutasteride-NRP1 may stem from the loss of interaction between dutasteride and two aromatic residues Trp^301^ and Tyr^353^ as shown in contact frequency analysis (Figure 4D). However, dutasteride has still a high affinity to the NRP1 and interacts with amino acids observed in docking simulations. Despite further in vivo analysis needed, dutasteride may be a preventive drug that shuts down the virus entry by blocking the NRP1 and downregulating the TMPRSS2. 

Antimigraine ergotamine is a tolerable drug with mild and transient side effects (Diener et al., 2002; Christie et al., 2003). Several in silico drug repurposing studies reported that ergotamine may inhibit the RdRp and 3CL^pro^ of SARS-CoV-2 (Barage et al., 2020; Gul et al., 2020). Ergotamine has the second-best BFE among all drugs analyzed in this study (Table 2). Comparable BFEs of ergotamine and EG01377 to NRP1 suggests that ergotamine can strongly bind to NRP1, in turn, can attenuate NRP1-protein S binding. If these computational analyses can be experimentally verified, ergotamine can be used to mitigate the COVID-19. To note, it has been reported that ergotamine may cross-react with antiviral drugs (Rosenthal et al., 1999; Mortier et al., 2001; Ayarragaray, 2014). Thus, further studies related to ergotamine should be designed accordingly.

Comparable BFE and interaction pattern of inhibitor and selected drugs indicate that these drugs may act as NRP1 inhibitors.

### 3.3. H-bond and Alanine scanning analysis

**Figure 5:**
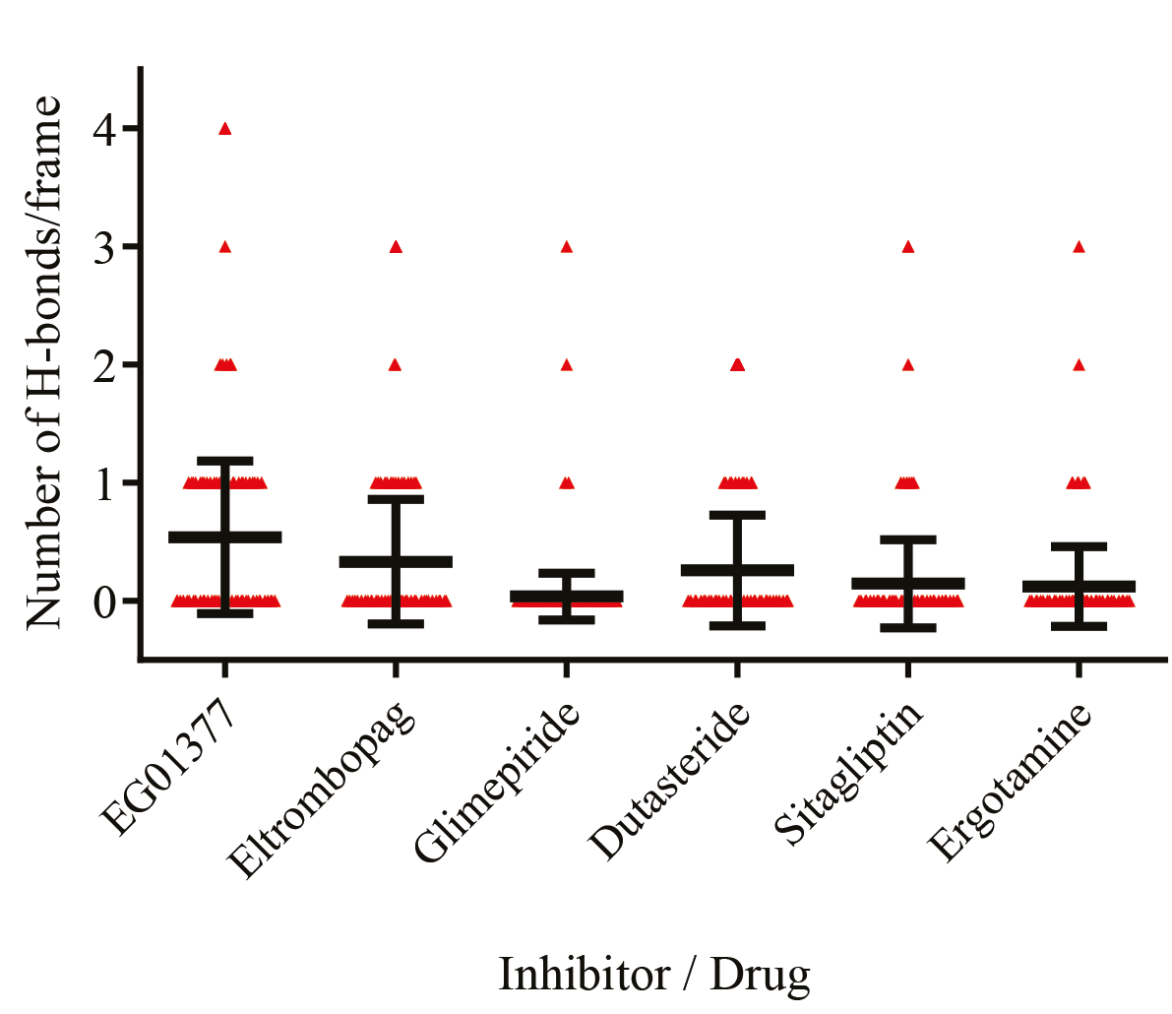
H-bond analysis of each drug/inhibitor made to NRP1 (per frame) in MD simulation. A total of 25,000 frames (every 4ps) were included for this calculation.

**Figure 6:**
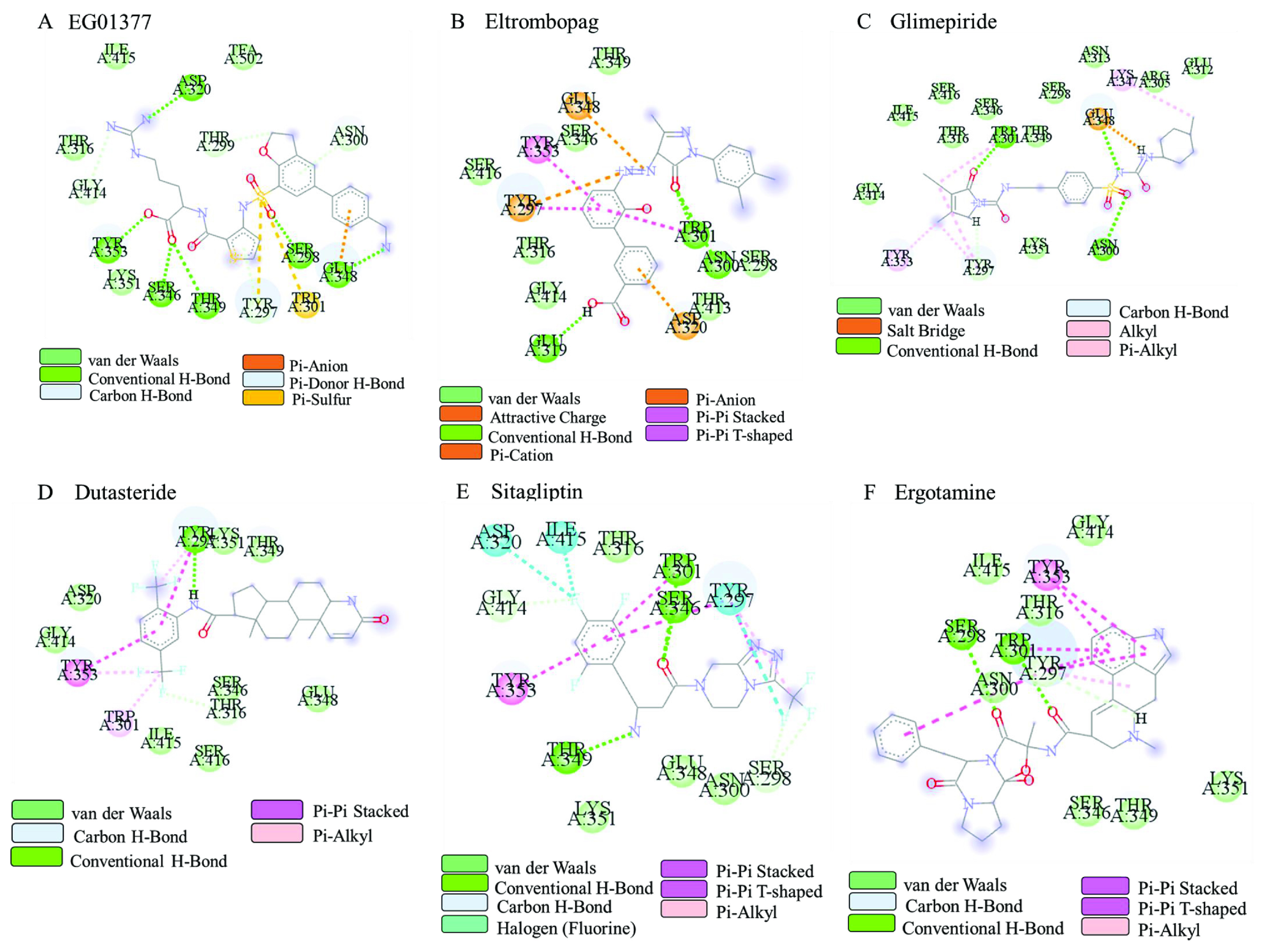
Two-dimensional diagram of interaction type between NRP1 in complex with (A) EG01377 in crystal structure (6FMF), or (B) eltrombopag, (C) glimepiride, (D) dutasteride, (E) sitagliptin, (F) ergotamine obtained from docking simulations.

To determine the number of hydrogen bonds drug made during the MD simulation, the hydrogen bond (H-bond) generated by drugs to any amino acids through 100 ns were analyzed (Figure 5). Calculation of the average number of H-bonds showed that each drug, including the inhibitor, produced less than one H-bond per frame. Interaction types between selected drugs and protein were analyzed and represented in a two-dimensional diagram (Figure 6). Analyses showed that the inhibitor and all drugs interact with the protein through hydrophobic interactions, and conduct hydrogen bond and at least one type of pi interaction. Since drugs of interest possess aromatic rings, charged or aromatic amino acids on NRP1 generate those interactions. Tyr^297^ consistently generated pi interactions, Trp^301^ and Tyr^353^ have generated either H-bond or pi interactions with all drugs. Ser^298^, Thr^316^, Glu^348,^ and Gly^414^ interacted with drugs through van der Waals interactions and generated hydrophobic interactions. Ser^298^ formed hydrogen bonds only with EG01377 and ergotamine. Glu^348^ generated pi interactions and hydrogen bonds with EG01377 and glimepiride, and only pi interactions with eltrombopag. 

Despite drugs suggested to behave like NRP1 inhibitor have diverse structures, they have some common properties. For example, all drugs analyzed in details have multiple ring structures at least one with aromatic. Second, all these organic compounds bear polar groups such as carbonyl group or halogen atoms that have ability to generate hydrogen bonds or pi interactions with amino acids. Third, drugs have 70 to 110 atoms in their structure that allow them to generate high number of van der Waals interaction with the protein. These common properties of drugs cause tight binding to NRP1.

**Figure 7:**
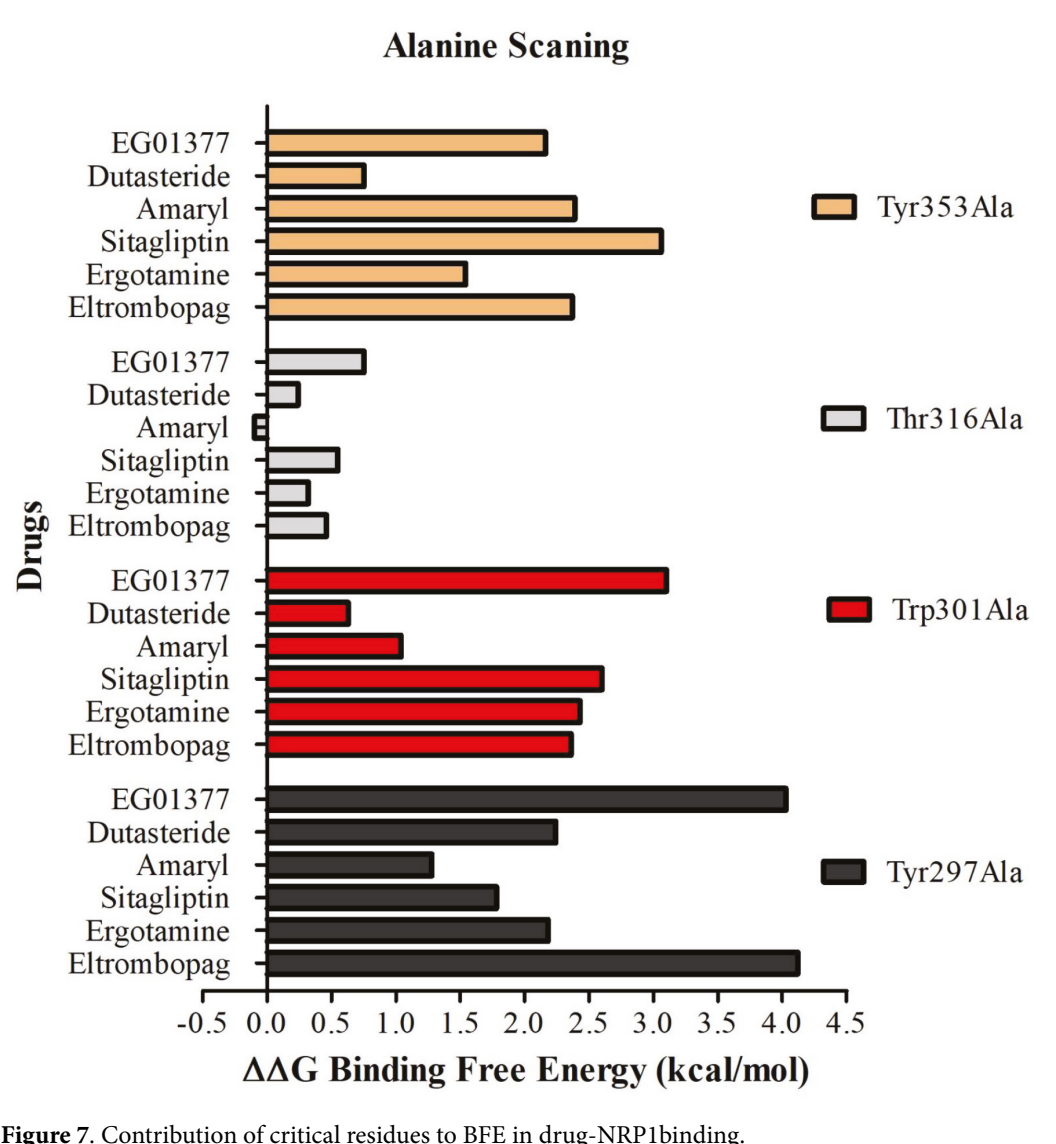
Contribution of critical residues to BFE in drug-NRP1binding.

To reveal the critical amino acids for drug binding and quantify their contribution to BFE, in silico Alanine scanning mutation was performed. The most frequently and commonly interacting amino acids e.g., Tyr^297^, Trp^301^, Thr^316^, and Tyr^353^ in all drug/inhibitor-NRP1 simulations were analyzed (Figures 4A–4F). These residues were mutated to Alanine one by one and BFE between drugs and NRP1 was calculated using the MM/GBSA method (Table 3). Then the difference between BFEs of drugs against mutant and wild-type NRP1 is calculated (ΔΔG). ΔΔG values larger than 0 means that mutation is destabilizing the interaction, ΔΔG lower than 0 means mutation is stabilizing the interaction. Free energy change in protein-protein interaction at equilibrium is calculated by using the following formula: ΔG^ᵒ^= –RTlnK_eq_ where ΔG^ᵒ ^is the standard free energy change, R is the gas constant, T is the absolute temperature. Using R = 1.987 × 10^-3^ kcal/mol, T = 298 K in that formula gives that increase in 0.4–0.5 kcal/mol in ΔG^ᵒ^ (ΔΔG in our data), leads to 2-fold decrease in ligand bound protein state. Thus, mutations causing ΔΔG > 0.5 kcal/mol were evaluated as critical amino acid for drug-protein binding. According to ΔΔG values, Tyr^297^, Trp^301^, and Tyr^353^ are very critical for all drugs binding to NRP1. Interestingly, despite high interaction with drugs, Thr^316^ minimally contributes to BFE. Tyr^297 ^has the highest contribution to BFE, –4.03, –4.12, –2.24 kcal/mol, in NRP1-EG01377, eltrombopag, and dutasteride simulations, respectively. Trp^301^ is the second highest contributor to BFE in EG01377 (–3.1 kcal/mol), eltrombopag (–2.36 kcal/mol), and sitagliptin (–2.60 kcal/mol) simulations and top contributor to BFE in ergotamine-NRP1 simulation (–2.43 kcal/mol). Tyr^353^ has a similar effect on the binding of eltrombopag to NRP1 with Trp^301^ and contributes –2.37 kcal/mol to BFE. Tyr^353^ has also the highest effect on BFE in sitagliptin (–3.06 kcal/mol) and glimepiride (–2.39 kcal/mol) simulations. (Table 3). The contribution of each mutated amino acid to drug-NRP1 BFE is given (Figure 7). 

H-bond and Alanine scanning calculations provide a deeper understanding of drug-NRP1 interactions. Future structure-activity relationship (SAR) studies aim to design a more potent NRP1 inhibitor may benefit from the critical amino acids identified here. Adding an H-bond donor or acceptor to a new molecule can increase the binding affinity to NRP1. 

**Table 3:** MM/GBSA BFE after Alanine mutations of selected residues.

	BFE (kcal/mol)			
Mutations Drugs	Y297A/ΔΔG	W301A/ΔΔG	T316A/ΔΔG	Y353A/ΔΔG
Eltrombopag	–12.99/4.12	–14.75/2.36	–16.65/0.46	–14.74/2.37
Ergotamine	–12.77/2.18	–12.52/2.43	–14.63/0.32	–13.41/1.54
Sitagliptin	–11.56/1.78	–10.74/2.60	–12.79/0.55	–10.28/3.06
Glimepiride	–11.27/1.28	–11.51/1.04	–12.65/–0.10	–10.16/ 2.39
Dutasteride	–7.23/2.24	–8.84/0.63	–9.23/0.24	–8.72/0.75
EG01377	–12.16/4.03	–13.09/3.1	–15.44/0.75	–14.03/2.16

## 4. Conclusion

Despite substantial progress has been made by several companies for the SARS-CoV-2 vaccine (Corbett et al., 2020; Mulligan et al., 2020; Sahin et al., 2020) only several governments approved for emergency use authorization. Therefore, medication is still an essential element to combat COVID-19. Vital enzymes of SARS-CoV-2 such as proteases (3CL^pro^ and M^pro^) and RNA polymerase (RdRp), or virus binding receptors on the host such as ACE2 or TMPRSS2 are generally targeted in drug repurposing studies (Bagheri and Niavarani, 2020; Busnadiego et al., 2020; Carino et al., 2020; Gul et al., 2020; Kumar et al., 2020; Li et al., 2020c; Singh et al., 2020b). Recent studies reported protein S binds to NRP1 which facilitates SARS-CoV-2 host entry (Cantuti-Castelvetri et al., 2020; Daly et al., 2020). Here a drug repurposing study was done against NRP1 in terms of docking and MD simulations using the FDA approved drugs. Furthermore, key amino acids on NRP1 for drug binding were identified by running Alanine scanning analysis. Several well-tolerated drugs showed comparable and even better affinity to NRP1 than its inhibitor EG01377. Eltrombopag had the best binding affinity to NRP1 among all drugs. In addition to eltrombopag, two antidiabetics, sitagliptin and glimepiride stably interacted with and exhibited high affinity to NRP1. Since patients having chronic diseases such as diabetes are at more risk against COVID-19, those suffering from diabetes may benefit from sitagliptin and glimepiride not only as antidiabetics but also their preventive effects, if proven by experiment, against SARS-CoV-2 infection. Dutasteride may provide multiple benefits to COVID-19 patients. First, it reduces the expression of TMPRSS2 that primes the S-protein (Hoffmann et al., 2020a; Hoffmann et al., 2020b). Second, in silico studies reported that it may block 3CL^pro^ (Gul et al., 2020). Third, this study shows that dutasteride can bind to and strongly interact with NRP1, thus, may decrease SARS-CoV-2 entry. Ergotamine is another top candidate molecule for the NRP1 binding. Previous calculations showed that ergotamine has the potential to bind to 3CL^pro^ and RdRp of SARS-CoV-2 (Gul et al., 2020; Rahman et al., 2020). Therefore, ergotamine may inhibit SARS-CoV-2 at the infection and replication stages. Alanine scanning calculations uncovered that Tyr^297^, Trp^301^, and Tyr^353 ^ are the most critical amino acid residues for drug binding to NRP1. H-bond analysis and Alanine scanning results may be exploited by further inhibitor design studies. 

To expedite the discovery of therapeutics for COVID-19 drug repurposing studies may provide a rational starting point. If the findings of this study can be verified by in vitro, in vivo, and clinical trials these well-tolerated and cost-effective drugs can be adapted to current protocols used to treat COVID-19.
